# Standard of Care and Outcomes of Primary Laparotomy Versus Laparotomy in Patients with Prior Open Abdominal Surgery (ReLap Study; DRKS00013001)

**DOI:** 10.1007/s11605-020-04904-z

**Published:** 2021-01-28

**Authors:** Dinh Thien-An Tran, Rosa Klotz, Julian C. Harnoss, Patrick Heger, Alina S. Ritter, Colette Doerr-Harim, Phillip Knebel, Martin Schneider, Markus W. Büchler, Markus K. Diener, Pascal Probst

**Affiliations:** 1 The Study Center of the German Surgical Society (SDGC), University of Heidelberg, Im Neuenheimer Feld 110, 69120 Heidelberg, Germany; 2 Department of General, Visceral and Transplantation Surgery, University of Heidelberg, Im Neuenheimer Feld 110, 69120 Heidelberg, Germany

**Keywords:** Primary laparotomy, Relaparotomy, Abdominal surgery

## Abstract

**Background:**

Patients undergoing relaparotomy are generally underrepresented in trials, despite how common the procedure is in clinical practice. The aim of this trial was to determine standard of care and gain evidence of intra- and postoperative outcomes for patients undergoing relaparotomy compared to primary laparotomy.

**Methods:**

In this single-center controlled clinical trial, adult patients scheduled for elective abdominal surgery via relaparotomy or primary laparotomy were consecutively screened for eligibility. The perioperative course was monitored prospectively in five study visits during hospital stay and one study visit 1 year after surgery. Intraoperative standards, short and long-term outcomes were statistically explored at a level of significance of 5%.

**Results:**

A total of 131 patients with relaparotomy and 50 patients with primary laparotomy were analyzed. In the relaparotomy group, the access to the abdomen took longer (23.5 min vs. 8.8 min; *p* = < 0.001) and the peritoneal adhesion index was higher (10.8 vs. 0.4; *p* = < 0.001). Inadvertent enterotomies were more frequent in the relaparotomy group (relaparotomy 0.3 versus primary laparotomy: 0.0; *p* = 0.002). The overall comprehensive complication index and rates of surgical site infection and wound dehiscence with evisceration were not different between the two groups. At long-term follow-up, rates of incisional hernia did not differ (relaparotomy: *n* = 12/104 (11.5%); primary laparotomy: *n* = 7/35 (20.0%); *p* = 0.208).

**Discussion:**

In this first prospective comparison of relaparotomy with primary laparotomy, inadvertent enterotomies were more frequent in the relaparotomy group. However, contrary to previous retrospective studies, the risk of complications and incisional hernias was not increased compared to primary laparotomy.

**Trial Registration:**

Deutsches Register Klinischer Studien (www.germanctr.de): DRKS00013001

**Supplementary Information:**

The online version contains supplementary material available at 10.1007/s11605-020-04904-z.

## Introduction

Although the minimally invasive approach to the abdominal cavity is becoming increasingly common, open incision (laparotomy) is still the standard surgical procedure and inevitable under various circumstances. In case of recurrent disease, a repeat laparotomy (relaparotomy) can be required in the further clinical course. Owing to advances in multimodal treatment and a higher life expectancy, today up to 66% of laparotomies in high-volume surgical centers are reoperations.^1^–^4^ Despite how common relaparotomy is in clinical practice, patients with relaparotomies are generally underrepresented in trials. Most multicenter randomized controlled trials and meta-analyses addressing abdominal wall closure after laparotomy exclude patients with relaparotomy.^5^–^7^

Current evidence suggests that adhesiolysis, which is frequently required in relaparotomies, increases the risk of inadvertent bowel injury, intraabdominal complications, wound infections and length of hospital stay.^1^,^8^ Besides, relaparotomy is suspected to pose a twofold risk of incisional hernia, resulting in higher costs and reduced quality of life.^9^,^10^

However, none of these trials prospectively compared the population of patients with relaparotomy to patients with primary laparotomy. Accordingly, an evidence-based approach to relaparotomy is not available. It is unclear to what extent the intraoperative standard of care in terms of surgical devices and materials used and time required for opening and closure of the abdominal wall differs between primary laparotomy and relaparotomy. Moreover, it is uncertain whether patients undergoing relaparotomy suffer from a higher risk of intraoperative complications (e.g., inadvertent enterotomies) or short- and long-term postoperative complications (e.g., wound infection or incisional hernia).

The aim of this trial was to close this gap of knowledge, and to gain evidence concerning the standard of care and perioperative outcomes in patients undergoing relaparotomy compared to those undergoing primary laparotomy.

## Methods

### Trial Design

ReLap was an investigator-initiated, single-center, prospective, mixed-methods (1st step: health care research, 2nd step: translational research, and 3rd step: randomized controlled trial) exploratory trial on patients undergoing relaparotomy.^11^ This is the report of the first step: a controlled clinical trial of patients undergoing relaparotomy versus primary laparotomy with the aim to gain evidence for the standard of care and intra−/postoperative outcomes of patients with relaparotomy. Step 2 including translational data from the same patients of step 1 on associations between biomarkers and adhesion grade has not been published, yet. Step 3, a randomized controlled trial on abdominal wall closure after relaparotomy has already been published elsewhere and reports the subset of the same relaparotomy patients from step 1 randomized to the small stitches technique, using Monomax 2–0, versus the large stitches technique, using PDS 1 loop.^12^ Supplemental figure 1 shows the flow of patients through all steps of the ReLap study. The trial was performed at the Clinical Trial Center of the Department of General, Visceral and Transplantation Surgery at the University of Heidelberg.

The ReLap trial was conducted in accordance with the current version of the Declaration of Helsinki,^13^ and according to the professional code for physicians in Germany (§15 BOÄ). The study protocol was reviewed and approved by the Ethics Committee of the medical faculty of the University of Heidelberg (S-442/2017). The protocol was published in a peer-reviewed open access journal.^11^

### Participants

All patients undergoing laparotomy were consecutively screened for eligibility. The following eligibility criteria were chosen to achieve a broad sample representative of high-volume surgical centers: Patients 18 years or older undergoing any kind of relaparotomy (status post any prior open abdominal surgery) or primary laparotomy were included. Patients undergoing relaparotomy for incisional hernia or laparostomy, undergoing an emergency operation or an operation of the retroperitoneum without transperitoneal access were excluded. Before inclusion in the ReLap study, patients were informed both orally and in writing about the study and gave their informed consent.

### Interventions

No study-specific interventions were performed. All patients received standard preoperative single-shot antibiotics, postoperatively antibiotics were only given therapeutically in case of infection. Laparotomy was routinely performed with an electric scalpel, alternatively surgical scissors and knives were used. Standard abdominal wall closure was achieved either with the small stitch technique using Monomax® 2–0 or with the large stitch technique using PDS II® 1-loop. In the relaparotomy group patients were randomized to these two techniques if the operating surgeon had no objection to randomization. However, the surgeon made the final decision regarding variations including additional sutures or mesh placement in case of clinically inapparent intraoperatively diagnosed hernia, diagnosed during surgery. In the primary laparotomy group the operating surgeon decided freely about the type of abdominal wall closure. Neither subcutaneous sutures nor subcutaneous drainages were placed routinely. The skin was closed with skin staples. All patients were treated within a standardized fast track concept, including physiotherapy-assisted early mobilization and early transition to a normal diet.

### Study Flow

The clinical course was followed prospectively. After preoperative screening (visit 1), intraoperative data was assessed by direct observation (visit 2), and the postoperative course was investigated on three postoperative visits until the day of definitive hospital discharge or postoperative day 30 (visit 3–5). The long-term follow-up was performed 1 year after surgery via a telephone visit (visit 6). If necessary, the patients’ general practitioners were also contacted. Patients and general practitioners were asked for the clinical occurrence of incisional hernia. No radiological proof was demanded. Presence of an incisional hernia, as well as whether or not the hernia required operative treatment was evaluated.

### Outcomes

Intraoperative outcomes included type of abdominal wall incision (electric scalpel or other device), length of skin and fascia incision, rate of incidental incisions of the rectus sheath, incidental enterotomies, and the presence of occult hernias. Furthermore, total time for opening (from skin incision to installation of the supporting frame) and closing of the abdominal wall (from final instrument count to skin closure), type of surgery and total operative time were assessed.

Existing adhesions were evaluated according to the peritoneal adhesion index, which divides the abdomen into 10 regions to be rated with a number from 0 (no adhesions) to 3 (strong adhesions) resulting in an index between 0 and 30.^14^ Overall postoperative morbidity was assessed according to the Clavien-Dindo classification^15^ and the comprehensive complication index.^16^ Wound dehiscence with evisceration, surgical site infection according to CDC definitions^17^ and postoperative hemorrhage were specifically assessed. Furthermore, time to first disposal of wind, time to first bowel movement, length of hospital stay, and length of stay on intensive care unit were evaluated. Long-term follow-up included quality of life (EuroQol five-dimensional questionnaire (EQ-5D)^18^ and incisional hernia rate.

### Sample Size

Given that ReLap was an exploratory study, no formal sample size calculation was performed for step 1. However, 100 patients with relaparotomy were deemed necessary to draw valid conclusions from step 3 (analysis of the subset of patients in the randomized controlled trial comparing the two techniques for abdominal wall closure^12^) to form hypotheses for future confirmatory trials. With a 2:1 ratio for the relaparotomy to primary laparotomy group, 50 consecutive patients undergoing primary laparotomy were included.

### Methods for Minimizing Bias

To avoid selective reporting, the study was registered with the German Clinical Trials Register (www.germanctr.de: DRKS00013001) before inclusion of the first patient. Randomization and blinding were obviously not possible due to the two different patient cohorts investigated. Statistical analysis was carried out after closure of the database according to a predefined analysis plan. The trial is reported according to the STROBE guidelines.^19^

### Statistical Methods

Data were presented either as mean with standard deviation or as rate. A descriptive *p* value was determined by chi-square test for binary data or Student’s *t* test for continuous data. Statistical analysis was performed with R.^20^

## Results

**Fig. 1:**
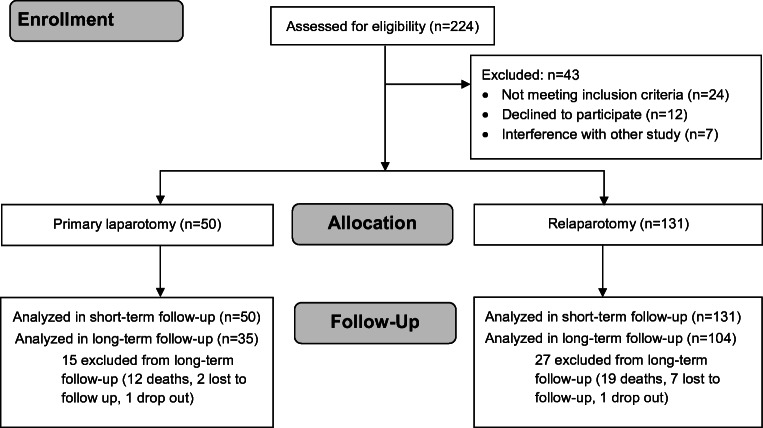
Study flowchart

Recruitment started on September 19, 2017, and the last patient was enrolled on April 4, 2018. Out of 224 patients assessed for eligibility, 43 patients were excluded as they did not meet inclusion criteria (*n* = 24), declined to participate (*n* = 12) or participated in other interventional studies (*n* = 7). For the randomized controlled part of this study (ReLap study step 3^12^), 100 patients with relaparotomies needed to be randomized to the two strategies of abdominal wall closure. Since some patients were not suitable for randomization, ultimately, 131 patients were recruited to the relaparotomy group. Thus, 131 patients in the relaparotomy group and 50 patients in the primary laparotomy group were analyzed. No patients were lost to short-term follow-up. A total of 104 patients (79.4%) in the relaparotomy group and thirty-five patients (70.0%) in the primary laparotomy group were available for long-term follow-up (Fig. 1).

### Baseline Data

Baseline characteristics of the study participants are presented in Table 1. The study population consisted of 74 women (40.9%) and 107 men (59.1%). The mean age was 62.0 ± 13 years, mean BMI was 24.6 ± 4.1 kg/m^2^, and mean Charlson comorbidity index was 2.5 ± 1.7. In the relaparotomy group, the most common indications for previous laparotomy were malignant disease (74.0%) and organ perforation (8.4%), and previous laparotomy was 1064 ± 1822 days ago. Gastrointestinal symptoms before surgery including nausea, emesis and obstruction were less frequent in the relaparotomy group. Some patients presented without any symptoms, whereas others reported various symptoms. Indication for current laparotomy was suspected malignant disease in 81.8%.

**Table 1:** Baseline characteristics

*n* (%) or mean (SD)	Relaparotomy (*n* = 131)	Primary laparotomy (*n* = 50)	*p* value*
Sex			
Female	53 (40.5%)	21 (42.0%)	0.984
Male	78 (59.5%)	29 (58.0%)	
Age (years)	60.1 (16.8)	65.1 (15.5)	0.076
BMI (kg/m^2^)	24.4 (3.9)	25.2 (4.4)	0.245
Charlson comorbidity index	2.5 (1.7)	2.7 (1.9)	0.440
ASA ≥ 3	56 (42.7%)	18 (36.0%)	0.511
Suspected malignancy as indication	104 (79.4%)	44 (88.0%)	0.260
Malign histology	81 (61.8%)	38 (76.0%)	0.105
Former radiotherapy	5 (3.8%)	1 (2.0%)	0.884
Former chemotherapy	49 (37.4%)	9 (18.0%)	**0.020**
Symptoms before surgery			
Abdominal pain	23 (17.6%)	13 (26.0%)	0.287
Nausea	16 (12.2%)	16 (32.0%)	**0.003**
Emesis	12 (9.2%)	11 (22.0%)	**0.038**
Obstruction	14 (10.7%)	12 (24.0%)	**0.041**
Infertility	0 (0.0%)	0 (0.0%)	
Other	9 (6.9%)	9 (18.0%)	0.050
Malign histology in previous operation	97 (74.0%)	n/a	n/a
Days since last operation	1064.5 (1822.1)	n/a	n/a

Differences with *P* values < 0.05 were deemed statistically significant and these *P* values are presented in bold

*Categorical variables: chi-square test; continuous variables: Student’s *t* test

*ASA*, American Society of Anesthesiologists; *n/a*, not applicable; *BMI*, body mass index

### Operative Data

All patients underwent elective operations. During primary laparotomy fascia was mainly incised with the electric scalpel (*n* = 48, 96.0%), whereas this technique was only used in half of the fascia incisions during relaparotomy (*n* = 64, 48.9%; *p* = < 0.001, Table 2). If the electric scalpel was not judged as safe enough, scissors were used. Access to the abdomen was achieved significantly slower in the relaparotomy group (23.5 ± 14.9 min) than in the primary laparotomy group (8.8 ± 4.1 min; *p* = < 0.001), and incidental incision of the rectus sheath occurred non-significantly more often in the relaparotomy group (relaparotomy: *n* = 29 (22.1%); primary laparotomy: *n* = 6 (12.0%); *p* = 0.182).

**Table 2:** Operative data

*n* (%) or mean (SD)	Relaparotomy (*n* = 131)	Primary laparotomy (*n* = 50)	*p* value*
Skin incision (cm)	26.7 (5.3)	25.7 (5.7)	0.264
Fascia incision (cm)	27.2 (5.1)	27.0 (4.6)	0.865
Abdominal fascia incision electric	64 (48.9%)	48 (96.0%)	**< 0.001**
Abdominal fascia incision scissors	67 (51.1%)	2 (4.0%)	**< 0.001**
Occult hernia	13 (9.9%)	2 (4.0%)	0.321
Incidental incision of the rectus sheath	29 (22.1%)	6 (12.0%)	0.182
Patients with adhesions	68 (51.9%)	3 (6.0%)	**< 0.001**
Peritoneal adhesion index	10.8 (8.2)	0.4 (1.1)	**< 0.001**
Patients with enterotomy	21 (16.0%)	2 (4.0%)	0.054
Average number of enterotomies	0.3 (0.8)	0.0 (0.2)	**0.002**
Access to the abdomen (min)	23.5 (14.9)	8.8 (4.1)	**< 0.001**
Operative time (min)	220.5 (112.9)	275.4 (115.9)	**0.005**
Abdominal wall closure (min)	26.4 (11.9)	26.0 (10.2)	0.807
Preparation fascia	57 (43.5%)	4 (8.0%)	**< 0.001**
Mesh placement	5 (3.9%)	0 (0.0%)	0.325
Intraabdominal drainage	84 (64.1%)	27 (54.0%)	0.280
Surgeon’s experience (years)	13.5 (7.3)	13.7 (8.1)	0.907
Resected organ systems**			
Liver	31 (23.7%)	12 (24.0%)	> 0.999
Pancreas	24 (18.3%)	31 (62.0%)	**< 0.001**
Stomach	18 (13.7%)	21 (42.0%)	**< 0.001**
Duodenum	17 (12.9%)	25 (50.0%)	**< 0.001**
Small intestine	56 (42,7%)	20 (40.0%)	0.868
Appendix	1 (0.8%)	1 (2.0%)	> 0.999
Colon	27 (20.6%)	7 (14.0%)	0.421
Gall bladder	16 (12.2%)	28 (36.0%)	**< 0.001**
Spleen	2 (1.5%)	6 (12.0%)	**0.008**
Kidney	4 (3.1%)	4 (8.0%)	0.297
Gynecologic	4 (3.1%)	1 (2.0%)	> 0.999
Central vessels	13 (9.9%)	9 (18.0%)	0.218
Peritoneum	18 (13.7%)	0 (0.0%)	**0.013**
Other	45 (34.3%)	7 (14.0%)	**0.011**

Differences with *P* values < 0.05 were deemed statistically significant and these *P* values are presented in bold

*Categorical variables: chi-square test; continuous variables: Student’s *t* test

**More than one organ was possible per operation

Adhesions were common in relaparotomy (*n* = 68 (51.9%)) and rare in patients undergoing primary laparotomy (*n* = 3 (6.0%); *p* = < 0.001). Accordingly, the peritoneal adhesion index was higher in relaparotomies (10.8 ± 8.2) than in primary laparotomies (0.4 ± 1.1; *p* = < 0.001). Accidental enterotomy during opening of the abdomen occurred non-significantly in more patients in the relaparotomy group (relaparotomy: *n* = 21 (16.0%); primary laparotomy: *n* = 2 (4.0%); *p* = 0.054); however, the overall number of enterotomies was significantly higher in the relaparotomy group (relaparotomy 0.3 ± 0.8; primary laparotomy: 0.0 ± 0.2; *p* = 0.002). The subgroup of patients with inadvertent enterotomies had a significantly higher peritoneal adhesion index compared to those patients without enterotomy (enterotomy: 16.0 ± 8.2; no enterotomy: 9.8 ± 7.9; *p* = 0.003).

Preparation of the fascia before abdominal wall closure was necessary in 57 patients (43.5%) during relaparotomy versus 4 patients (8.0%) during primary laparotomy (*p* = 0.001). Occult hernia of the abdominal wall was found in 13 patients during relaparotomy (9.9%) and 2 patients during primary laparotomy (4.0%; *p* = 0.321) requiring mesh placement in 5 patients in the relaparotomy group (3.8%). Duration of abdominal wall closure showed no difference between groups (relaparotomy: 26.4 ± 11.9 min; laparotomy: 26.0 ± 10.2 min; *p* = 0.807).

Length of skin incision, length of fascia incision, drain placement and surgeon’s experience did not differ between the two groups. Overall length of surgery was longer in the primary laparotomy group.

### Postoperative Outcomes

Postoperative outcomes are presented in Table 3. The CCI did not differ between groups (relaparotomy: 17.1 ± 20.3; primary laparotomy: 18.4 ± 18.2; *p* = 0.638). Frequency of surgical site infection showed no difference between the groups, irrespective of whether the wound infection was superficial, deep, or at organ space. Rate of wound dehiscence with evisceration, other complications (graded according to the Clavien-Dindo classification), length of hospital stay and length of intensive care unit stay were similar between groups. Duration until first bowel movement was longer in the primary laparotomy group and more patients received laxative medication. The rate of incisional hernia after 1 year and necessity for treatment showed no significant difference.

**Table 3:** Postoperative outcomes

*n* (%) or mean (SD)	Relaparotomy (*n* = 131)	Primary laparotomy (*n* = 50)	*p* value*
Complications according to Clavien-Dindo classification			
I	62 (0.47 pp)	32 (0.64 pp)	**0.045**
II	64 (0.48 pp)	26 (0.52 pp)	0.705
IIIa	15 (0.11 pp)	9 (0.18 pp)	0.245
IIIb	19 (0.14 pp)	10 (0.20 pp)	0.367
IVa	0 (0.00 pp)	1 (0.02 pp)	0.108
IVb	0 (0.00 pp)	0 (0.00 pp)	> 0.999
V (mortality)	3 (2.3%)	0 (0%)	0.281
CCI	17.1 (20.3)	18.4 (18.2)	0.638
Surgical site infection total	43 (32.8%)	14 (28.0%)	0.532
Superficial	23 (17.5%)	7 (14.0%)	0.565
Deep	4 (3.1%)	1 (2.0%)	0.699
Organ/space	16 (12.2%)	6 (12.0%)	0.969
Wound dehiscence with evisceration	3 (2.3%)	1 (2.0%)	0.906
Postoperative hemorrhage	10 (7.6%)	3 (6.0%)	0.953
Postoperative laxative medication	99 (75.6%)	46 (92.0%)	**0.023**
Time until first bowel movement (days)	2.5 (1.5)	3.0 (1.4)	**0.045**
Time until first wind disposal (days)	2.4 (1.5)	2.6 (1.6)	0.317
Length of hospital stay (days)	12.1 (8.9)	14.8 (13.3)	0.176
Length of ICU stay (days)	1.4 (5.1)	3.3 (12.3)	0.278
Incisional hernia at 1 year**	12/104 (11.5%)	7/35 (20.0%)	0.208
In need for surgical therapy**	6/104 (5.8%)	1/35 (2.9%)	0.679

Differences with *P* values < 0.05 were deemed statistically significant and these *P* values are presented in bold

*CCI*, comprehensive complication index; *pp*, per patient

*Categorical variables: chi-square test; continuous variables: Student’s *t* test

**Data for 35 patients in primary laparotomy group and 104 patients in relaparotomy group

The subgroup of patients with inadvertent enterotomy presented with a comparable length of hospital stay as patients without enterotomy (enterotomy: 15.4 ± 8.2; no enterotomy: 11.4 ± 9.8; *p* = 0.196). The CCI was non-significantly higher in patients with inadvertent enterotomy than in patients without enterotomy (enterotomy: 25.6 ± 24.0; no enterotomy: 15.5 ± 19.2; *p* = 0.080).

### Quality of Life

The scale between 0 and 100 for overall quality of life did not differ between the two groups at the preoperative visit, at the time of discharge or 1 year after surgery. Preoperatively, more patients in the primary laparotomy group reported some problems concerning mobility when compared with the relaparotomy group (24.0% versus 9.9%; *p* = 0.027). None of the five dimensions of the EQ-5D differed at the other study visits (day of discharge and 1 year after surgery). Preoperative quality of life and quality of life at the day of discharge were comparable between the two groups, and better than quality of life 1 year after surgery. Table 4 gives a detailed overview of quality of life.

**Table 4:** Quality of life

Quality of life EuroQol five-dimensional questionnaire	Preoperative			Hospital discharge			One year		
*n* (%) or mean (SD)	Relaparotomy (*n* = 131)	Primary laparotomy (*n* = 50)	*p* value*	Relaparotomy (*n* = 131)	Primary laparotomy (*n* = 50)	*p* value*	Relaparotomy (*n* = 104)	Primary laparotomy (*n* = 35)	*p* value*
Mobility									
No problems	118 (90.1%)	38 (76.0%)	**0.027**	101 (77.1%)	34 (68.0%)	0.186	81 (77.1%)	28 (80.0%)	0.884
Some problems	13 (9.9%)	12 (24.0%)		28 (21.4%)	13 (26.0%)		21 (20.2%)	6 (17.1%)	
Many problems	0 (0.0%)	0 (0.0%)		2 (1.6%)	3 (6.0%)		2 (1.9%)	1 (2.9%)	
Independence									
No problems	123 (93.9%)	47 (94.0%)	> 0.999	108 (82.4%)	34 (68.0%)	0.107	88 (84.6%)	30 (85.7%)	0.908
Some problems	8 (6.1%)	3 (6.0%)		19 (14.5%)	13 (26.0%)		14 (13.5%)	4 (11.4%)	
Many problems	0 (0.0%)	0 (0.0%)		3.1%)	3 (6.0%)		2 (1.9%)	1 (2.9%)	
Daily tasks									
No problems	94 (71.8%)	40 (80.0%)	0.065	49.6%)	15 (30.0%)	0.054	49 (47.1%)	19 (54.3%)	0.749
Some problems	35 (26.7%)	7 (14.0%)		55 (41.9%)	28 (56.0%)		47 (45.2%)	14 (40.0%)	
Many problems	2 (1.5%)	3 (6.0%)		11 (8.4%)	7 (14.0%)		8 (7.7%)	2 (5.7%)	
Pain									
No problems	79 (60.3%)	30 (60.0%)	> 0.999	23 (26.0%)	27 (34.0%)	0.301	51 (49.0%)	19 (54.3%)	0.756
Some problems	44 (33.6%)	17 (34.0%)		25 (50.0%)	23 (26.0%)		43 (41.3%)	12 (34.3%)	
Many problems	8 (6.1%)	3 (6.0%)		2 (4.0%)	0 (0.0%)		10 (9.6%)	4 (11.4%)	
Fear									
No problems	75 (57.3%)	36 (72.0%)	0.062	78 (59.5%)	29 (58.0%)	0.899	52 (50.0%)	21 (60.0%)	0.271
Some problems	47 (3.6%)	10 (20.0%)		45 (34.4%)	17 (34.0%)		43 (41.3%)	11 (31.4%)	
Many problems	9 (6.9%)	4 (8.0%)		8 (6.1%)	4 (8.0%)		19 (18.3%)	3 (8.6%)	
Overall	63.6 (21.8)	62.8 (21.9)	0.818	61.2 (22.6)	62.2 (24.9)	0.841	49.6 (32.0)	45.9 (36.3)	0.528

Differences with *P* values < 0.05 were deemed statistically significant and these *P* values are presented in bold

*Categorical variables: chi-square test; continuous variables: Student’s *t* test

## Discussion

The absence of standardization of surgical and perioperative care and non-compliance with current guidelines is common and has been criticized.^21^ This is the first prospective study to describe specific clinical practice including surgical techniques for opening and closure of the abdomen and the additional time required during relaparotomy compared to primary laparotomy. However, previous retrospective studies suggested higher rates of postoperative wound infection, wound dehiscence with evisceration and incisional hernia upon relaparotomy.^1^,^8^–^10^ Specifically, this clinical controlled trial was performed to evaluate the standard of care on the one hand and the risk for postoperative complications on the other hand of relaparotomy compared to primary laparotomy.

In relaparotomy, due to more frequent and severe adhesions, laparotomy took longer compared to primary laparotomy, and fascia incision could only be performed safely with the electric scalpel in half of the cases. Despite great care and using scissors instead, accidental enterotomy during laparotomy was more frequent in the relaparotomy group. However, this trial suggests a comparable risk for postoperative complications including wound infections, postoperative hemorrhage, wound dehiscence with evisceration and incisional hernia, as well as comparable quality of life after relaparotomy and primary laparotomy.

The described worsening of the quality of life 1 year after surgery in both groups can most likely be explained by progression or current treatment (e.g., chemotherapy) of underlying oncological disease as > 80% of patients suffered of malignant diseases. However, the exact oncological status was not specifically evaluated in the long-term follow-up.

Patients with enterotomies were non-significantly more frequent in the relaparotomy group, had a higher peritoneal adhesion index than patients without inadvertent enterotomies, and presented with a non-significantly, but clinically relevant higher CCI. Non-significance of these results can most likely be attributed to a too small sample size. Thus, the risk for postoperative complications is most likely increased after incidental intraoperative enterotomy.

The overall surgical site infection rate in this trial (32%) was rather high compared to available literature investigating patients undergoing primary laparotomy (4–37%)^5^,^22^–^24^ and relaparotomy (6.5–12%).^9^ However, if the organ/space surgical site infections are excluded and only superficial and deep surgical site infections are considered, the rates are in line with literature. Furthermore, differences in the definition of surgical site infections on the one hand and the presence of several risk factors for surgical site infections in our study population such as comorbidities, oncologic resections and extended, mostly contaminated resections with prolonged operative times on the other hand likely have contributed to higher rates.^22^,^23^,^25^

Obviously, two different groups were compared and it was one of the aims of this trial to assess the differences between relaparotomy and primary laparotomy patients. Specifically, the two study populations differ in the following aspects: Less patients in the relaparotomy group reported gastrointestinal complications and impaired mobility preoperatively. This may be due to the fact that patients undergoing relaparotomy are followed up regularly in oncological care programs. Therefore, indications for relapse surgery may be given earlier and, thus, prior to the onset of symptoms as compared to patients with primary diagnosis of malignant disease. Besides, average duration of surgery was shorter and pancreatic resection was less frequent in the relaparotomy group compared to the primary laparotomy group, which might be caused by the performance of major surgical procedures in the primary laparotomy group. Furthermore, time until first bowel movement was shorter and postoperative use of laxative medication was less frequent in the relaparotomy group. These aspects suggest that patients in the primary laparotomy group might have been in worse condition than in the relaparotomy group. On the other hand, more patients in the relaparotomy group had undergone chemotherapy before surgery. All these factors may influence the risk of perioperative complications. Also, other unknown covariates may have confounded the results. In future trials comparing patients with relaparotomy versus primary laparotomy propensity matching might be useful to assure comparability of the study groups.

This clinical controlled trial has several limitations. No formal sample size calculation has been performed, and the low numbers of cases might lead to the absence of significant differences in postoperative complications, which might be different in a confirmatory multicenter setting. Broad inclusion criteria were chosen to achieve a patient population representative for a high-volume general surgery center. Nonetheless, the monocentric design might impair external validity of the study results as it cannot be safely assumed that the standards described here apply elsewhere.

Besides, for 1 year follow-up with assessment of incisional hernia rates the patient and his general practitioner were called by phone. Personal clinical or radiologic assessment of a potential incisional hernia was not performed since the visit was done as a telephone interview. Therefore, the rate of incisional hernias is limited to clinically obvious hernias. However, clinically relevant hernias causing discomfort should be identifiable with this method and 1-year rates of incisional hernia are comparable to previous multicenter trials.^5^ Finally, the follow-up of 1 year for assessment of incisional hernia is short and results may not adequately describe the final incisional hernia rate, as the European Hernia society recommends a follow-up of at least 2 years.^26^ Both length of follow-up and the pragmatic approach for assessment were chosen because of the exploratory nature of the presented trial but would not be adequate in a confirmatory setting.

To summarize, this prospective comparison of relaparotomy with primary laparotomy describes and quantifies differences in standard of care between relaparotomy and primary laparotomy for the first time. It shows that during relaparotomy, scissors instead of electric scalpel were used more frequently, laparotomy took longer and inadvertent enterotomies were more prevalent. The increased risk of wound infections, wound dehiscence with evisceration und incisional hernias in relaparotomy proposed in previous mostly retrospective literature was not confirmed in this prospective trial: the risk for postoperative complications and incisional hernias did not differ between relaparotomy and primary laparotomy.

## Supplementary Information

Supplemental figure 1 Patient flow through all steps of the ReLap study as published in the protocol^12^ (PNG 591 kb)

High resolution image (TIF 124 kb)
