# Supplementary figures and images for: Standard of Care and Outcomes of Primary Laparotomy Versus Laparotomy in Patients with Prior Open Abdominal Surgery (ReLap Study; DRKS00013001)
Source: J Gastrointest Surg. 2021 Jan 28;25(10):2600–9. doi: 10.1007/s11605-020-04904-z (PMC8523469; doi:10.1007/s11605-020-04904-z)

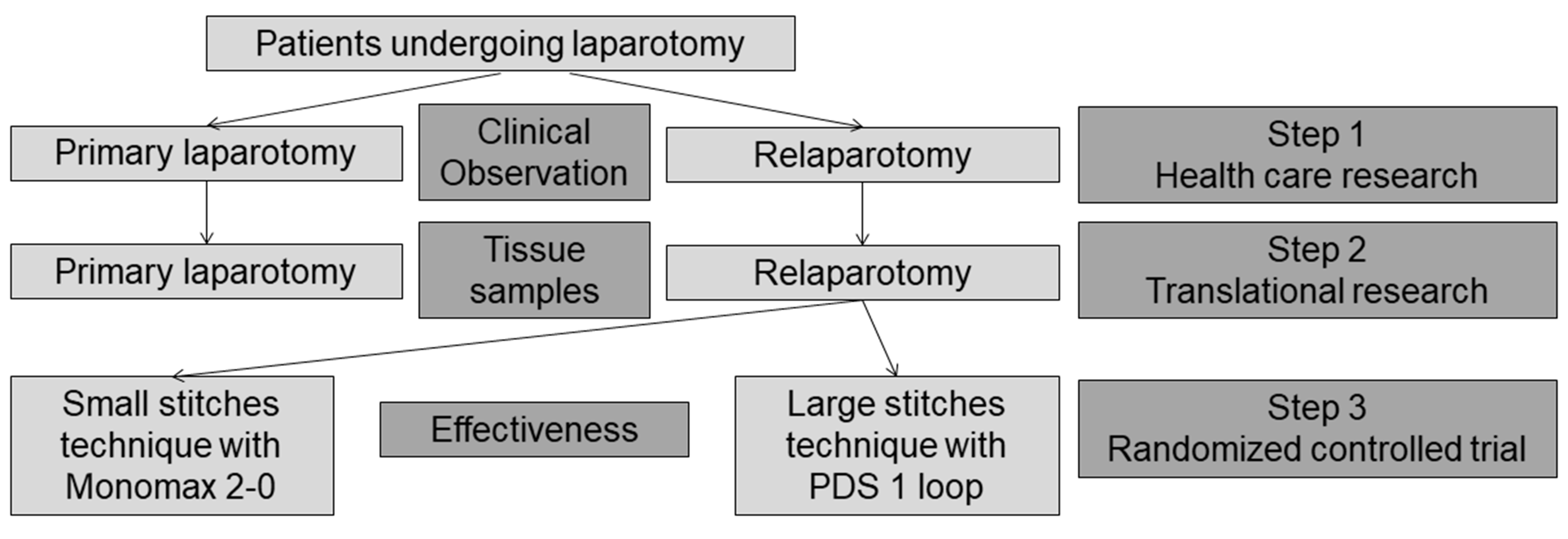

Supplement: Supplementary file 1 — Patient flow through all steps of the ReLap study as published in the protocol12 (PNG 591 kb) [file 11605_2020_4904_Fig2_ESM.png]

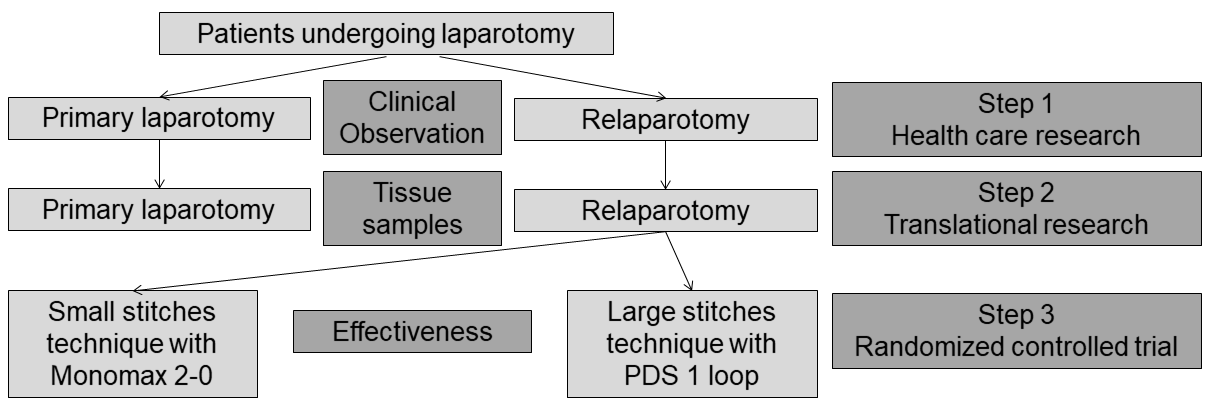

Supplement: Supplementary file 2 — High resolution image (TIF 124 kb) [file 11605_2020_4904_MOESM1_ESM.tif]
